# Efficacy of an Ethanol-Based Hand Sanitizer for the Disinfection of Blood Pressure Cuffs

**DOI:** 10.3390/ijerph16224342

**Published:** 2019-11-07

**Authors:** Lucia Grandiere Perez, Céline Ramanantsoa, Aurélie Beaudron, Cyril Hoche Delchet, Pascale Penn, Pauline Comacle

**Affiliations:** 1 Service de Maladies Infectieuses et Tropicales, Centre Hospitalier, 194 avenue Rubillard, 72000 Le Mans, France; 2 Laboratoire de Microbiologie, Centre Hospitalier, 194 avenue Rubillard, 72000 Le Mans, France; cramanantsoa@ch-lemans.fr (C.R.); abeaudron@ch-lemans.fr (A.B.); choche@ch-lemans.fr (C.H.D.); ppenn@ch-lemans.fr (P.P.); pauline.comacle@gmail.com (P.C.)

**Keywords:** blood pressure cuff, sphygmomanometer, hand sanitizer, hand rub, ethanol, disinfection

## Abstract

Blood pressure cuffs (BP cuffs) have been implicated in some nosocomial outbreaks. We compared the efficacy of an ethanol-based hand sanitizer (EBHS) with a detergent/disinfectant for the disinfection of BP cuffs. The inner sides of 30 BP cuffs were sampled for bacterial culture. Then, the same area was divided into halves. One half was disinfected by a detergent/disinfectant and the other was disinfected by an EBHS. The bacterial count decreased significantly with both disinfectants (*p* < 0.0001 compared with before disinfection). The bacterial count decrease seemed greater with the EBHS compared with the detergent/disinfectant, but the difference was not significant. Therefore, within the limits of a single application, the EBHS was an efficacious means of BP cuff disinfection. However, the repeated exposure to emollients contained in EBHS may require further studies before validating these results.

## 1. Introduction

As with many other patients with cardiovascular risk, patients with chronic kidney disease check their blood pressure very often, to reduce the complications of high blood pressure. Regrettably, blood pressure cuffs (BP cuffs) are implicated in nosocomial infections, with potentially high clinical and economic costs [1,2,3,4,5]. 

Most guidelines recommend BP cuff disinfection between each patient with a detergent/disinfectant [6], but the disadvantage is skin irritation; the health care worker should wear gloves, a time consuming and not inexpensive task. In common practice in many hospitals, disinfection of BP cuffs between each patient is not performed [1,2,7,8]. 

Improving environmental disinfection can reduce health-care associated infections, notably those due to methicillin-resistant *Staphylococcus aureus* (MRSA), vancomycin-resistant enterococci, *Acinetobacter baumanii* and *Clostridium difficile-*associated diarrhea [9]. In a Japanese hospital, improving the frequency of disinfection of BP cuffs with alcohol was followed by a decline in MRSA cuff contamination [8]. Similarly, in an American dermatology ward, improving the disinfection of blood pressure cuffs was followed with a reduction of contamination of BP cuffs and of nosocomial infections with MRSA and borderline methicillin-susceptible *Staphylococcus aureus* [5].

Ethanol based hand sanitizers (EBHS) include an emollient to protect the skin. They are well tolerated and easily available in hospital wards, but their efficacy in disinfecting BP cuffs has not been evaluated. The aim of this study is to compare the efficacy of an EBHS and of a detergent/disinfectant for the disinfection of BP cuffs.

## 2. Materials and Methods

Thirty nylon BP cuffs, used daily, from 14 medical units of a general hospital (Le Mans Hospital, France) were analyzed. The 14 units were medicine wards (1 polyvalent consultation and 13 hospitalization wards: nephrology, pneumology, cardiology, infectious diseases, neurology, geriatric service, rheumatology, polyvalent medicine). In the hospitalization wards, each nurse made a “blood pressure tour” per day, for 11 patients on average. As there are three nurse shifts per day in each ward, each BP cuff was used on average 30 times per day. 

The recommended practice is to disinfect the cuff with a detergent/disinfectant after each measure, but the common practice is to disinfect the BP cuff after it has been used on a carrier of multiresistant bacteria, on immunosuppressed patients, and at the end of the “blood pressure tour”. The nurses disinfect the BP cuffs with the detergent/disinfectant, they wear gloves, pour the detergent/disinfectant on a cloth, and rub the inner surface of the BP cuff.

For the study, we sampled with a swab (ESwab^TM^, Copan) an area of 10 cm × 10 cm, in the inner side of each BP cuff. Then, this area was divided into halves. One half was disinfected with 2 mL of detergent/disinfectant (didecyldimethylammonium chloride and polyhexamethylene biguanide chlorhydrate, Surfa’safe^R^, Anios, France) and the other half was disinfected with 2 mL of an EBHS (ethanol 700 mg/g 755 mL/l N°CAS 64-17-5, water and emollient agents, Aniosrub 85 (Non Parfumé non Coloré)NPC^R^, Anios France). Once fully dry (10 min), each half area was sampled. Samples were cultured using tryptone soy agar with sheep blood plates (BioMérieux^®^) and chromogenic media for detection of *Staphylococcus aureus* (MRSA), extended-spectrum beta-lactamase (ESBL)-producing enterobacteria, and vancomycin-resistant enterococci (VRE). After 48 h incubation at 37 °C, bacteria colony forming units (CFU) were quantified and the two dominant species were identified by mass spectrometry with a matrix-assisted laser desorption ionization time-of-flight (MALDI-TOF, Bruker, Germany).

The comparisons of bacteria CFU counts between groups (“before disinfecting” versus “after disinfecting with the detergent/disinfectant”; “before disinfecting” versus “after disinfecting with the EBHS” and “detergent/disinfectant” versus “EBHS”) were made by a non-parametric test (Wilcoxon for paired samples), taking into account the Bonferroni correction, considering that we performed three tests.

## 3. Results

Each BP cuff was disinfected three times a day in 57% of the units, and once a day in the remaining units (43%).

Most of the bacteria found (55/60 = 91.7%) were potential pathogens. These included cutaneous bacteria (coagulase-negative *Staphylococci* 77%, including *Staphylococcus lugdunensis* 1.7%; MRSA *3.3%*), bacteria derived from the mucosae (*Enterococcus faecalis* 3.3%, *Moraxella* sp. 1.7%, *Corynebacterium aurimucosum* 1.7%), and from the environment (*Acinetobacter johnsonii* and *lwoffii* 5%). Non-pathogenic bacteria (8.3%) included *Micrococcus luteus* (5%) and *Bacillus* sp. (3.3%) (Table 1).

**Figure 1:**
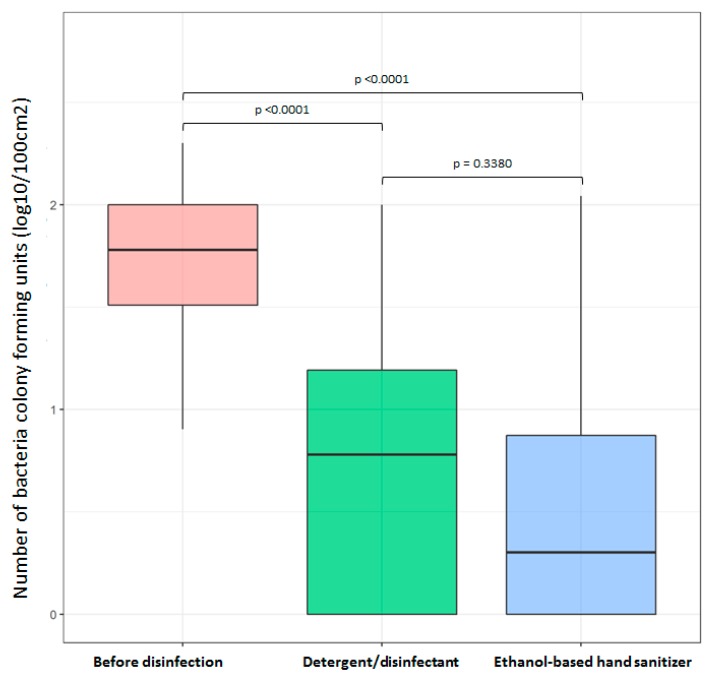
Number of bacteria colony forming units on 30 blood pressure cuffs, before and after disinfection with the detergent/disinfectant or the ethanol-based hand sanitizer.

Before disinfection, the average bacteria CFU was 82 CFU/100 cm^2^. After disinfection with the detergent/disinfectant and the EBHS, the average bacteria CFU was 16 and 10 CFU/100 cm^2^, respectively (Figure 1). The Wilcoxon non-parametric test for paired samples showed a significant bacterial decrease with the EBHS and the detergent/disinfectant (*p* < 0.0001 for each). The bacterial count decrease seemed greater with the EBHS compared with the detergent/disinfectant, but the difference was not significant (*p* = 0.338). After disinfection, the virulent bacteria MRSA, *Staphylococcus lugdunensis,* and *Enterococcus faecalis* were no longer isolated. 

The number of bacteria colony forming units is presented in log10 per 100 cm^2^ (range 0 to 2.3).

Statistical analyses show a significant (*p* < 0.0001) decrease of bacteria counts with the detergent/disinfectant and the ethanol hand sanitizer (Wilcoxon non-parametric test). The difference between the detergent/disinfectant and the hand sanitizer is not significant (*p* = 0.338).

## 4. Discussion

Our study suggests that using an EBHS for BP cuff disinfection is associated with a significant decrease in the number of potentially pathogenic bacteria. There was no evidence that the EBHS was better than the detergent/disinfectant. Similarly, a Japanese study found that an 80% ethanol solution was efficacious in disinfecting BP cuffs which were contaminated by MRSA, but the study was focused only on MRSA and not on other bacteria [8]. Interestingly, some studies have shown an efficacy of hand sanitizers to disinfect stethoscopes [10,11].

The main bacteria identified on our 30 BP cuffs were coagulase-negative staphylococci (75% of all identified bacteria); other authors found this predominance too, with 8% to 45% of BP cuffs contaminated [1,12]. Sixteen percent of our BP cuffs were contaminated by virulent bacteria: MRSA (6.7%), *Enterococcus faecalis* (6.7%), and *Staphylococcus lugdunensis* (3.3%, Table 1). Other authors have found virulent bacteria on BP cuffs; these included MSSA (up to 33% [1,12,13]), MRSA (up to 31% [1,8,13,14]), *Clostridium difficile* (10% to 33% [2,13]), and vancomycin-resistant enterococci (up to 18% [3,4]). During the investigation of nosocomial outbreaks, several studies have found genetic links between bacteria from infected patients and cultures from BP cuffs for *S. aureus* [1,5], VRE [3,4], *Pseudomonas aeruginosa*, and *Serratia marcesens* [1]. 

Most of these bacteria may cause life threatening infections like bacteremia, endocarditis (MRSA, *Staphylococcus lugdunensis*, *Enterococcus faecalis*), pneumonia (*Pseudomonas aeruginosa*), severe diarrhea (*Clostridium difficile)*, and device-related infections (coagulase negative staphylococci, MSSA, MRSA). This may be of particular importance in immunocompromised patients, such as many patients with chronic kidney diseases, and in patients with medical devices like prostheses, cardiac devices, and catheters, as is the case for many dialysis patients. 

The downside of the EBHS is that it contains emollients. The routine use of such solutions would lead to the build-up of a sticky residue on the surface of the cuffs, necessitating periodic washing. Furthermore, it is not known if the accumulation of the emollient could interfere with EBHS efficacy. Further research may be addressed to test the disinfecting efficacy of the EBHS after repeated applications on BP cuffs.

Within the limits of a reduced sample size, and of testing only one application of the EBHS or of detergent/disinfectant, our study has the merit of driving attention on a neglected aspect of daily care, BP cuff disinfection, and, in a broader sense, of making us reflect on the importance of simple maneuvers in infection control in medical wards. 

## 5. Conclusions

Our study, within the limits of a small sample size and a single application, suggests that EBHS may be an efficacious means for BP cuff disinfection. In hospitalization wards, hand sanitizer is usually more easily available and is better tolerated than detergents/disinfectants. However, the repeated exposure to emollients contained in EBHS may require further study before validating these results.

## Figures and Tables

**Table 1:** Bacteria species identified on the 30 blood pressure (BP) cuffs (two dominant species by BP cuff).

Bacteria Species Identified on the 30 BP cuffs (Two Dominant Species by BP cuff)		
Clinical Impact of the Bacteria	Bacteria Species	Percentage of Identified Bacteria
Virulent bacteria (5/60 = 8.3%)	Methicillin-resistant *Staphylococcus aureus*	2/60 = 3.3%
	*Enterococcus faecalis*	3.3%
	*Staphylococcus lugdunensis*	1.7%
Potentially pathogenic bacteria * (50/60 = 83.3%)	coagulase-negative staphylococci	75%
	*Acinetobacter johnsonii*	3.3%
	*Acinetobacter lwoffii*	1.7%
	*Corynebacterium aurimucosum*	1.7%
	*Moraxella sp.*	1.7%
Usually non-pathogenic bacteria (5/60 = 8.3%)	*Micrococcus luteus*	5%
	*Bacillus*	3.3%

* Potentially pathogenic bacteria may cause device-related infections and infections in immunocompromised patients.
